# Evening types have social jet lag and metabolic alterations in school-age children

**DOI:** 10.1038/s41598-020-73297-5

**Published:** 2020-10-07

**Authors:** Nuria Martínez-Lozano, Gloria Maria Barraco, Rafael Rios, Maria José Ruiz, Asta Tvarijonaviciute, Paul Fardy, Juan Antonio Madrid, Marta Garaulet

**Affiliations:** 1 Department of Physiology, University of Murcia, IMIB-Arrixaca, CP 30100 Murcia, Spain; 2 Health Area of Lorca, Lorca, Murcia Spain; 3 Division of Sleep and Circadian Disorders, Departments of Medicine and Neurology, Brigham and Women’s Hospital, Boston, MA 02115 USA

**Keywords:** Biomarkers, Risk factors

## Abstract

Chronotype has been mostly assessed with subjective scales. Objective assessment has been undertaken with actigraphy, although problems may occur in classifying chronotype. The aims of the study were to assess chronotype in school-age children using a novel integrative measurement (TAP) derived from non-invasive assessments of wrist temperature (T) physical activity (A) and body position (P) and to explore associations between chronotype, sleep disturbances, and metabolic components. Four-hundred-thirty-two children of 8–12 years were recruited from a Mediterranean area of Spain. Measurements were: (a) Chronotype objectively (7-day-rhythms of TAP) and subjectively measured (Munich-chronotype-self-reported questionnaire); (b) sleep rhythms and light exposition; (c) 7-day-diaries of food intake; (d) anthropometry and metabolic parameters; (e) academic scores. TAP acrophase was able to assess eveningness. As compared to more morning-types, more evening-types displayed lower amplitude in temperature rhythms, increased physical activity in the evening, delayed sleep and midpoint of intake and had more frequent social jet lag (*P* < 0.05). More evening-types had higher light intensity at 2 h before sleep and lower melatonin values (01:00 h). Eveningness associated with higher BMI and metabolic risk (higher values of insulin, glucose, triglycerides and cholesterol). Evening-types presented better grades in art. In conclusion, more evening-types, as objectively assessed, presented sleep alterations, social jet lag, obesity and higher metabolic risk.

## Introduction

Chronotype is a characteristic that helps to determine circadian typology^1,2^. Differences in the relationship between an individual’s circadian phase and external local time result in chronotypes that range from early to late^3^. Early chronotypes tend to perform better in the morning while late chronotypes perform better in the evening^3^.

In adults, evening chronotype is associated with health complications^4^, lower physical activity, short sleep duration and social jet lag^5,6^. Studies performed at earlier ages in different chronotypes are scarce and less is known about school aged children, particularly those that relate unhealthy behaviors and metabolic risk^7^.

Questionnaires are widely used to assess individual chronotype^8^. Nevertheless, the accuracy of such questionnaires depends on self-reporting, good recall, and the subject’s ability to complete them correctly and honestly^9^. Therefore, the use of objective tools capable to capture individual chronotype in a simple, continuous, and non-invasive form in free living conditions, is necessary, particularly in children at school ages.

Continuous monitoring of physical activity by actigraphy has been used as an objective assessment of chronotype in free living conditions, although studies have been mostly performed in pre-school children^10^ and adolescents^11^ but not in school-age children. Furthermore, actigraphy has problems in measuring midpoint of sleep because it tends to overestimate sleep and underestimate wake time^12^. Including wrist temperature may be of benefit because it is considered as a good sleep marker^13^. A recent consensus document sponsored by the National Heart Lung and Blood Institute, National Institute on Aging and the Sleep Research Society^14^, has stated that wrist temperature is a novel and less invasive method of measuring circadian phase timing and sleep and wake states.

TAP is an integrative variable that combines wrist temperature (T), physical activity (A) and body position (P) and has been shown to be a powerful method to assess individual chronotype, circadian system status and sleep characteristics in adults^15^. Compared with conventional actigraphy, TAP has been shown to be clinically superior in evaluating sleep objectively^16^. It improves sensitivity, specificity, and accuracy when compared with physical activity, body position or body temperature alone, and it minimizes masking effects such as those derived from environmental temperature or from device failures^15^. TAP has been validated in healthy and unhealthy subjects^17^ with dim light melatonin determinations (DLMO) and with polysomnography, in determining chronotype and circadian health^18^. It has also been shown that TAP, together with other non-invasive tools, is able to assess circadian health in children^19^. However, no studies exist evaluating the utility of TAP to determine chronotype in school-age children.

The purpose of this study is to assess TAP as a novel integrative measurement to determine chronotype and sleep patterns in school-age children and to study whether objectively assessed evening chronotypes show increased metabolic risk, social jet lag and sleep alterations as compared to morning-types.

## Methods

### Subjects

Four hundred thirty-two healthy children ages 8 to 12 years were recruited from three schools in a Mediterranean area of Spain between October 2014, and June 2016 (ClinicalTrials.gov ID: NCT02895282) as already described^19^. Approval for this study was obtained by the Ethics Committee of the University of Murcia. Written consent to participate was provided by the parents. All procedures performed in studies involving human participants were in accordance with the ethical standards of the institutional and/or national research committee and with the 1964 Helsinki declaration and its later amendments or comparable ethical standards. Recruitment procedure and methodology have been previously described^19^.

### TAP derived chronotype and sleep variables (Table 1)

Subjects wore a wristwatch during 7 days of study, on the non-dominant hand, that integrated a wrist temperature sensorcollecting information every 5 min, and an accelerometer sensor that measured physical activity and body position every 30 s as previously described^19,20^. From these measures the TAP algorithm was calculated^15^. Individual chronotype and sleep parameters were obtained from TAP as follows^15^.

**Table 1:** Sleep and circadian-related variables.

Chronotype	Abreviations	Definition
Acrophase		Time period during which the daily cycle of TAP peaks
Objective chronotype		Acrophase of TAP determined by Cosinor’s analysis
Subjective chronoype	MCTQ	Individual chronotype assesed by Munich Chronotype Questionnaire
**Sleep parameters**		
Central sleep timing		Timing of the average of the five consecutive hours of maximum values of sleep
Circadian Function Index	CFI	A numerical index that determines the circadian robustness, based on three circadian parameters: Interday Stability (IS), Intraday Variability (IV) and Relative Amplitude (RA). CFI oscillates between 0 (absence of circadian rhythmicity) and 1 (robust circadian rhythm)
Day–night contrast		Difference between the average of measurements for the five consecutive hours with the maximum TAP and the average of measurements made for the 10 consecutive hours with the minimum TAP divided by the sum of both values
Depth of sleep		Hourly average during the 5 consecutive hours of minimum values of TAP
Duration of sleep		Difference between sleep bedtime and sleep awake time
Interday stability	IS	Constancy of the 24 h rhythmic pattern over days. A stable rhythm is characterized by a 24 h profile that remains very similar from day to day
Intraday variability	IV	Fragmentation of the rhythm. Its values oscillate between 0 when the wave is perfectly sinusoidal and 2 when the wave describes a Gaussian noise
Regular habits		Derived from the Interday stability (IS): determines the constancy of the 24 h rhythmic pattern over the 7 days. A stable rhythm is characterized by a 24 h profile that remains very similar from day to day
Relative amplitude	RA	Difference between the maximum (or minimum) value of the cosine function and mesor
Social jet lag		Difference in the midpoint of sleep between weekend (MSFsc) and weekdays (MSW); (Social jet lag = MSFsc—MSW). Subjects with more than 2 h of difference in the midpoint of sleep between weekend and weekdays were identified as having social jet lag^21^
Midpoint of food intake		Average of the seven days of the midpoint between breakfast and dinner times (first and last eating episode)
TAP algorithm	TAP	The integrated TAP variable is calculated using the following procedure: we first normalized the TAP variables by calculating the 95th and 5th percentiles for each variable. Wrist temperature values were inverted since activity and position values were opposites, so that the maximum values for all 3 variables occurred at the same time of the day. Then we calculated the mean of the 3 normalized variables, where 0 corresponded to complete rest and sleep and 1 to periods of high arousal and movement

#### Individual chronotype

Acrophase of TAP determined by Cosinor’s analysis was used as an objective biomarker of the individual chronotype (Table 1). More evening-types, neither-types and more morning-types were classified by the acrophase´s tertiles (higher values for the evening-types). An age appropriate Spanish version of the Munich Chronotype Questionnaire (MCTQ) was used to subjectively determine individual chronotype^2^. The MCTQ was designed to measure sleep times separately for work and free days and to estimate chronotype based on the time-based variable of the MCTQ. The midpoints of sleep were calculated for weekend (Free days) (MSF) and Weekdays (MSW). MSF was corrected as follows: MSFsc = MSF − 0.5 × (SDF − (5 × SDW + 2 × SDF)/7), where SDF was sleep duration on free days and SDW was sleep duration on work days^21^. Social jet lag was calculated as the difference between mid-sleep on free days and mid-sleep on work days as follows Social jet lag = MSFsc − MSW. Subjects with more than 2 h of difference in the midpoint of sleep between weekend and weekdays were identified as having social jet lag.

#### Sleep parameters

A 0 value of TAP indicated complete rest, whereas a 1 value corresponded to wakefulness and movement. An epoch was scored as sleep when TAP was under a default threshold, previously validated by polysomnography^22^. Time in movement, determined as the time in which a movement on any of three axes was detected, was used to discriminate between sleep and wake states.

From TAP the following sleep characteristics were determined by non-parametric analyses: sleep duration, circadian function index^23^, interdaily stability (IS), relative amplitude (RA), central sleep timing, depth of sleep, regular habits and day–night contrast (Table 1).

#### Light exposition

A *luxmeter* was programmed to collect light information continuously every 30 s. Subjects were instructed to wear the luxmeter on a lanyard over their clothing and to place it on a bedside table when asleep, as previously described^24^.

#### Daytime physical activity

Average physical activity in wakefulness was obtained from the 7-day activity record.

#### Food timing

A 7-day dietary record was completed that included food quantities and timing, and the midpoint of food intake.

#### BMI and waist circumference

BMI and waist circumference measurements were collected on the first day of the week of study as already described^19^.

#### Saliva and serum determinations

Melatonin was determined by radioimmunoassay (IBL, Germany) from two salivary samples one at night (01:00 h) and one before lunch (14:00 h). Glucose, insulin, cholesterol and triglycerides were determined from serum and saliva samples by conventional methods (Beckman Coulter Ireland Inc., Ireland).

#### Academic performance

Academic performance of a subpopulation was collected (n = 92). Grades for each subject were determined from overall performance on tests, as well as knowledge demonstrated during the academic year. Grades were assessed in Spanish language, mathematics, natural sciences, social sciences, English, French, artistic education, physical education and catholic religion and an average score was calculated.

#### All statistical analyses

All statistical analyses were performed using SPSS version 20.0 (SPSS, Chicago, Illinois, USA). Values of *P* < 0.05 were considered to be statistically significant. Differences between more morning-type, neither-type and more evening-type were analyzed by ANCOVA adjusted for gender, age, race, academic year, BMI and total energy intake (Table 2). In addition, Pearson correlation analyses were performed between (1) TAP acrophase and circadian characteristics (Table 2) and (2) TAP and metabolic parameters (Table 3). Linear regression was also used to test for associations between chronotype and metabolic parameters. Further adjustments for initial BMI and total energy intake were performed (Partial correlation analyses). Biomarkers in saliva and serum were log-transformed in base 10.

**Table 2:** Differences between morning, neither and evening chronotypes in circadian-related variables and academic performance.

	Individual chronotype			*P*(1)	*P*(2)	*P*(3)	Correlation (4)	
	Morning-type	Neither-type	Evening-type				r	*P*
	(n = 141)	(n = 141)	(n = 144)					
Girls (%)	48.6	44.7	58.3	0.059	0.037*	0.021**		
**Characteristics**	Mean ± SD	Mean ± SD	Mean ± SD					
Age (year)	10 ± 1.18a	10 ± 1.21a	10 ± 1.34a	0.623	0.871	0.979	0.068	0.164
**Chronotype markers**								
*Objective assessment*								
TAP acrofase (hh:mm)	14:26 ± 00:19a	15:08 ± 00:10b	15:54 ± 00:25c	< 0.001	< 0.001	< 0.001		
Melatonin at 01:00 h (pg/ml)	29.88 ± 21.26a	25.03 ± 13.97b	24.79 ± 17.14b	0.030	0.038	0.070	− 0.124	0.013
*Subjective assessment*								
MCTQ (hh:mm)	3:50 ± 0:37a	4:03 ± 0:36b	4:12 ± 0:44c	< 0.001	< 0.001	< 0.001	0.225	< 0.001
**Midpoint of food intake (hh:mm)**	14:56 ± 0:16a	15:03 ± 0:20b	15:11 ± 0:22c	< 0.001	< 0.001	< 0.001	0.319	< 0.001
**Daytime activity (%)**	206.32 ± 28.87a	206.73 ± 25.69a	198.06 ± 28.17b	0.015	0.008	0.074	− 0.151	0.002
**Regular habits (%)**	91.49 ± 15.00a	93.38 ± 15.41a	85.85 ± 18.42b	< 0.001	< 0.001	0.004	0.339	< 0.001
**Light exposition**								
Light acrophase (hh:mm)	13:55 ± 0:22a	14:20 ± 0:20b	14:43 ± 0:24c	< 0.001	< 0.001	< 0.001	0.677	< 0.001
Light during the day (log lux)	2.20 ± 0.45ab	2.35 ± 0.21a	2.12 ± 0.10b	0.033	0.059	0.066	− 0.163	0.072
Light before bed time (log lux)	0.29 ± 0.19a	0.35 ± 0.19ab	0.42 ± 0.19b	0.022	0.030	0.048	0.285	0.002
**Sleep variables**								
*Duration*								
Sleep duration (hh:mm)	9:29 ± 0:38a	9:20 ± 0:35ab	9:11 ± 0:42b	0.001	0.007	0.001	− 0.169	0.001
Short sleepers (n (%))	3(1)	4(1)	14(4)	0.003	0.067	0.087		
*Circadian Function Index (CFI)*	0.82 ± 0.08a	0.84 ± 0.05b	0.81 ± 0.10a	0.007	0.006	0.011	− 0.080	0.099
Relative amplitude (RA)	0.96 ± 0.11ab	0.99 ± 0.03a	0.94 ± 0.16b	0.007	0.007	0.009	− 0.144	0.003
Interdaily stability (IS)	0.67 ± 0.14a	0.71 ± 0.12b	0.66 ± 0.15a	0.006	0.005	0.018	− 0.061	0.212
*Sleep characteristics*								
Central sleep timing (hh:mm)	3:20 ± 1:14a	3:30 ± 1:10a	4:13 ± 1:07b	< 0.001	< 0.001	< 0.001	0.356	< 0.001
Depth of sleep (%)	82.30 ± 12.94a	80.33 ± 18.72ab	76.76 ± 23.25b	0.044	0.039	0.020	− 0.128	0.008
Day–night contrast (%)	90.41 ± 13.83ab	91.78 ± 12.35a	88.02 ± 15.77b	0.074	0.081	0.221	− 0.150	0.002
**Social jet lag**								
Social jet lag (hh:mm)	1:12 ± 0:40a	1:19 ± 0:38ab	1:29 ± 0:45b	0.003	0.039	0.010	0.167	0.001
Social jet lag n (% of children)	12 (3)	14 (4)	26 (7)	0.001	0.002	0.001		
**Academic performance**								
Arts score	5.96 ± 1.48a	6. 69 ± 1.20b	6.84 ± 1.08b	0.024	0.050	0.293	0.264	0.011
Average score	7.71 ± 1.29a	7.63 ± 1.34a	7.70 ± 1.11a	0.244	0.481	0.628	0.172	0.097

(1) Differences among chronotypes assessed by ANOVA; (2) Differences among chronotypes assessed by ANCOVA adjusted for sex, age, race, academic year and BMI. (3) Differences among chronotypes assessed using ANCOVA adjusted for sex, age, race, academic year, BMI, and total energy intake. (4) Pearson’s correlation between TAP acrophase and circadian-related variables. *Differences among chronotypes assessed using ANCOVA adjusted for age, race, academic year and BMI; ** Differences among chronotypes assessed using ANCOVA adjusted for age, race, academic year; BMI and total energy intake. Different letters indicate significant differences among chronotypes. MCTQ: Munich Chronotype Questionnaire. Social jet lag = MSF – MSW > 2 h.

**Table 3:** Correlation between acrophase of TAP and metabolic parameters.

	n	r	*P (1)*	*P(2)*	*Β* (1)	SEM(1)	*P (1)*	*Β* (3)	SEM(3)	*P(*3)
Serum cholesterol (mg/dl)	73	0.311	0.007	0.008	0.043	0.016	0.007	0.040	0.016	0.016
Serum triglycerides (mg/dl)	73	0.313	0.008	0.001	0.074	0.027	0.008	0.090	0.025	0.001
Saliva insulin (µUI/mL)	125	0.242	0.007	0.001	0.195	0.071	0.007	0.179	0.077	0.021
Saliva glucose (mg/dl)	126	0.250	0.005	0.002	0.472	0.166	0.005	0.383	0.176	0.032
BMI (kg/m^2^)	424	0.099	0.041		0.578	0.282	0.041	0.368	0.178	0.041^#^
Body fat of girls (%)*	174	0.168	0.027	0.238	1.748	0.784	0.027	− 0.434	0.308	0.161

(1) Pearson’s correlation test; (2) Adjusted by BMI. (3) Adjusted by BMI and total energy intake.

*Boys did not show significant differences. Biomarkers in saliva and serum were log-transformed in base 10.

^#^Adjusted by total energy intake.

## Results

### TAP as a marker of chronotype

**Figure 1:**
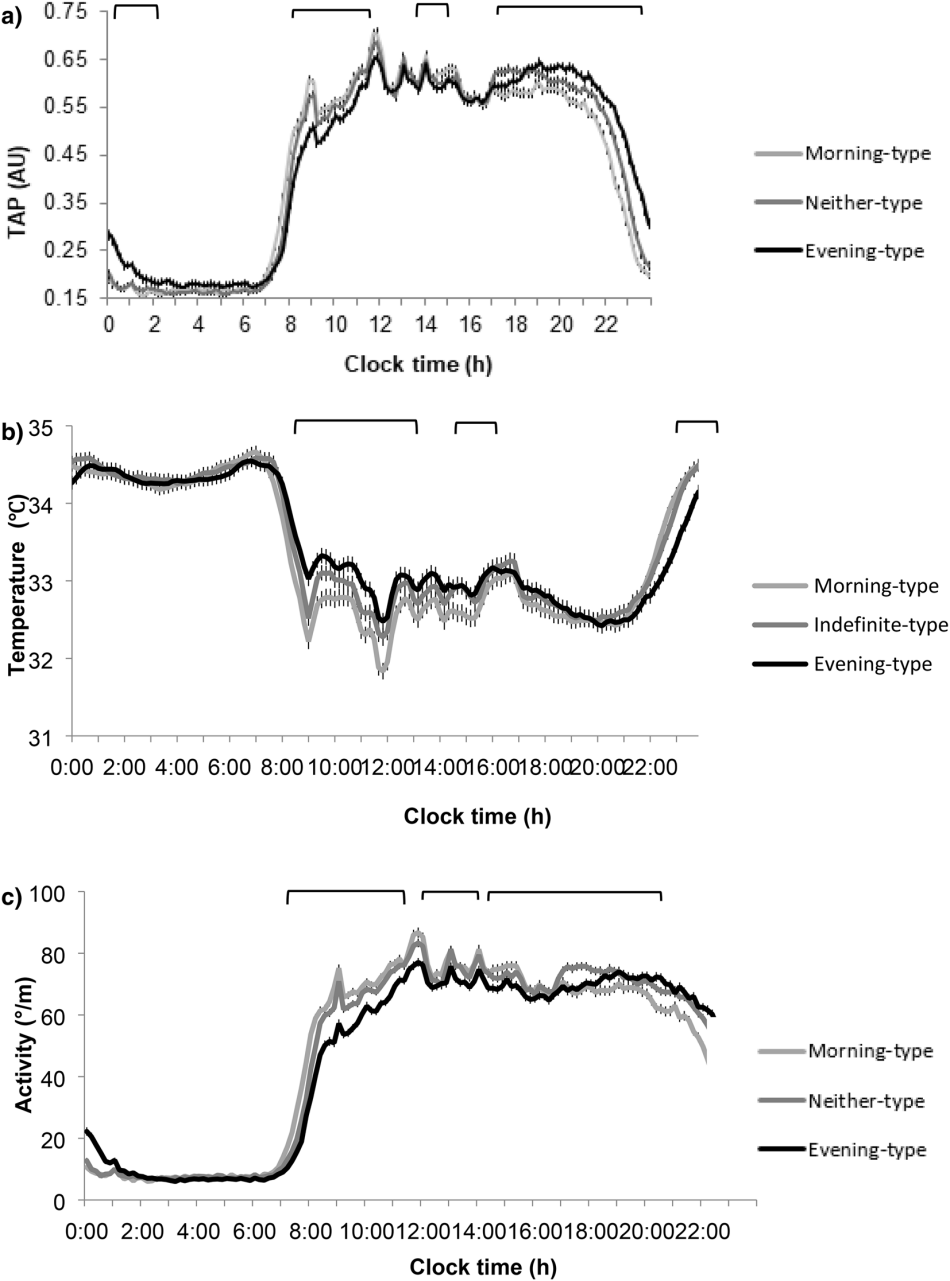

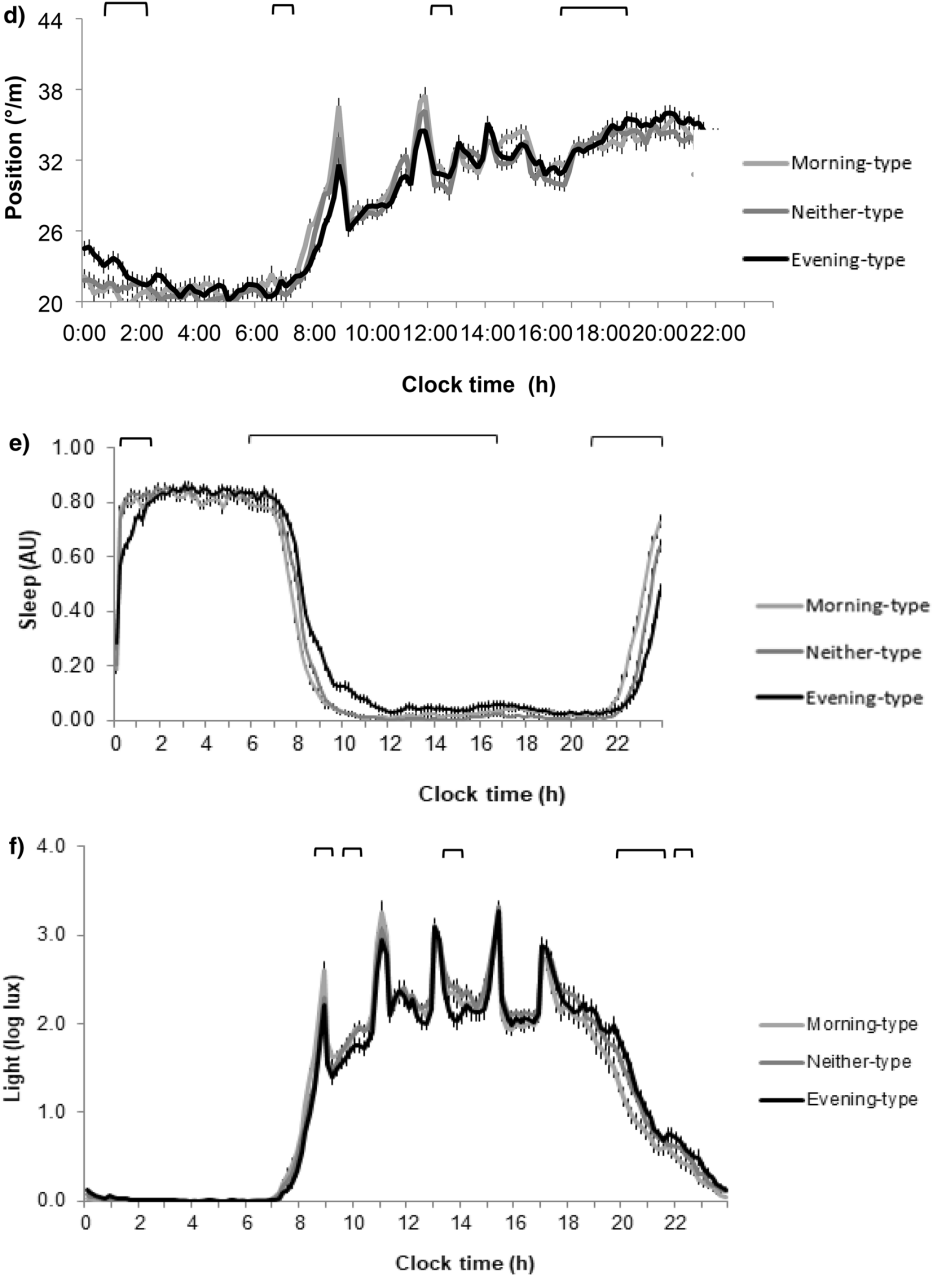
Average daily patterns recorded over a seven-day period of (**a**) Integrative variable TAP (from peripheral temperature, activity and position) (n = 432) divided in chronotypes by tertiles, (**b**) temperature, (**c**) activity, (**d**) position, (**e**) sleep in the total population of (n = 432) and (**f**) light exposition in a subpopulation (n = 120) in morning, neither and evening chronotypes children. Differences among chronotypes was assessed by ANOVA. The upper brackets represents the hours at which the pattern differs significantly (*P* < 0.05).

Seven-day rhythms of TAP (Fig. 1a) differed among the three objectively classified chronotypes (more morning, neither and more evening). As compared to more morning types, evening types showed a delayed pattern of TAP and lower values in the morning and higher in the evening (Fig. 1a). Similarly, subjective chronotype (derived from the Munich questionnaire), central sleep timing and midpoint of food intake, were also significantly delayed in more evening-types as compared to more morning-types (Table 2) and subjective and objective chronotypes correlated significantly with one another (r = 0.225; *P* < 0.001). As expected, saliva melatonin levels at 01:00 h were lower in more evening than in more morning-types (*P* < 0.05) (Table 2). Melatonin decreased by 3.43 (95% CI 5.963 to 0.902) pg/ml *per* hour of later chronotype (*P* = 0.008). These data suggest that TAP acrophase was correctly classifying the three independent chronotypes.

More evening-types had higher values of body temperature in the morning (more sleepiness) and lower at night (more awakeness) than morning types (Fig. 1b). By contrast, evening-types had lower values of physical activity and body position during the first morning hours and higher values during the evening (*P* < 0.05) (Fig. 1c,d). In general, day-time physical activity was lower in evening-types as compared to neither-types and morning-types (*P* < 0.05) (Fig. 1c, Table 2).

### Sleep characteristics

**Figure 2:**
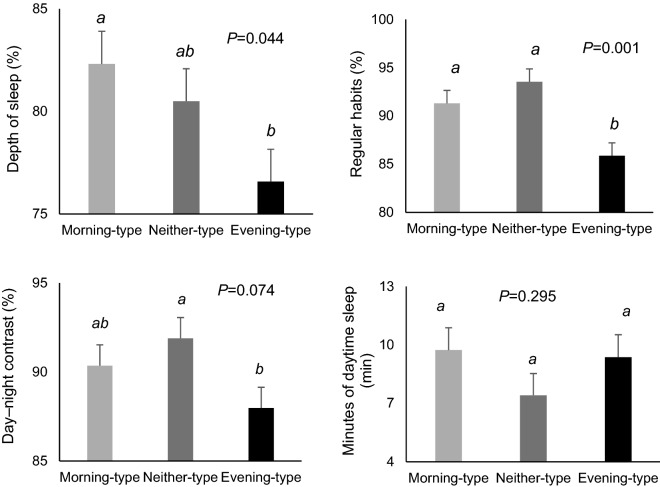
Differences between morning-type, neither-type and evening-type in sleep characteristic and regular habits. Differences among chronotypes are indicated in the graphs with the Post-hoc-value of ANOVA. Different superscripts mean significant differences (*P* < 0.05).

Habitual sleep duration was 09:19 ± 0:39 h. Six percent of subjects were short sleepers (duration less than 8 h) and 12% had social jet lag, (more than 2 h of difference between weekdays and weekends). Daily patterns of sleep of all subjects are presented in Fig. 1e. Delayed sleep occurred in evening-types, with higher levels of sleepiness during the day, mainly during the first hours, although sleepiness was still significantly higher until 16:00 h (*P* < 0.05). Evening-types had shorter sleep duration and the proportion of short sleepers was 4 times greater in evening-types than morning-types (Table 2). Evening-types had lower sleep circadian function index *(P* = 0.007) with decreased relative amplitude (*P* = 0.007) and lower interday stability (*P* = 0.006). Depth of sleep and day–night contrast was also decreased in evening types (Fig. 2), who showed less regular habits than the other chronotypes.

### Social jet lag

The differences between the weekday and weekend midpoint of sleep was 17 min higher in evening-types than in morning-types (Table 2). Furthermore, evening-types experienced social jet lag more frequently than morning-types, 7% and 3%, respectively (*P* = 0.001).

### Light exposure

**Figure 3:**
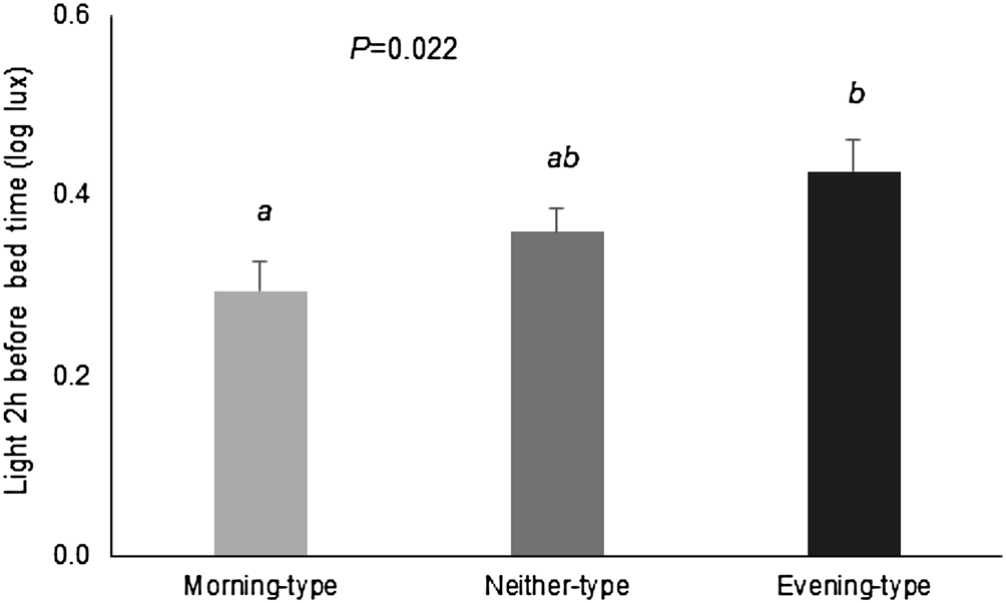
Differences in light 2 h before bed time of morning, neither and evening chronotype children. Differences among chronotypes are indicated in the graphs with the Post-hoc-value of ANOVA. Different superscripts mean significant differences (*P* < 0.05).

The light pattern was delayed approximately 1 h in evening-types (acrophase) (Table 2). Total light intensity was lower at daytime and higher at nighttime (Fig. 1f). Later light acrophase was associated with 0.85 (95% CI 0.41 to 1.29) hours later of TAP acrophase, and therefore with a later chronotype (*P* = 0.001). The light intensity, at 2 h before sleep timing, i.e. the timing in which melatonin starts to rise was 31% higher in more evening-types than in more morning-types (Fig. 3).

### Obesity and metabolic risk

**Figure 4:**
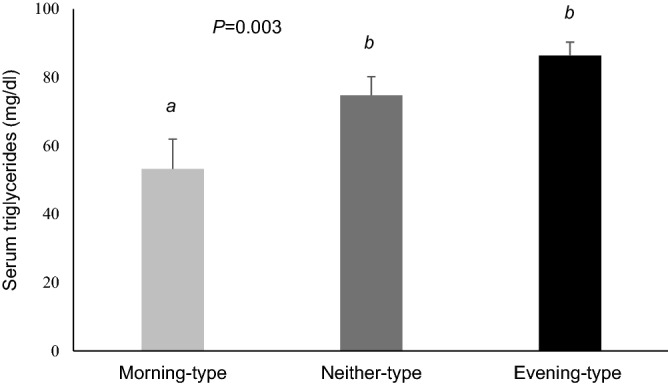
Differences in the triglycerides values of morning, neither and evening chronotype children. Differences among chronotypes are indicated in the graphs with the Post-hoc-value of ANOVA. Different superscripts mean significant differences (*P* < 0.05).

Evening-type was associated with higher BMI and higher metabolic risk markers such as glucose, insulin, cholesterol and triglycerides levels (Table 3 and Fig. 4). A delay of 1 h in the chronotype was related with a 0.56 increase in BMI (*P* = 0.036). Associations with metabolic risk markers were still present after adjusting for BMI and habitual energy intake.

### Academic performance

Evening types had significantly higher scores in art (*P* = 0.024) (Table 2), although there were no significant differences when adjusted for BMI and habitual energy intake.

## Discussion

One aim of the present study was to assess TAP as a measure of chronotype in school age children. TAP simultaneously considers circadian endogenous wrist temperature and variables which are more dependent on willingness such as habitual physical activity and body position^9,15^. Because TAP is a non-invasive test used in free living conditions it is particularly suitable for this age group. Results from the study provide insight into evening chronotype and its association with sleep alterations, social jet lag, obesity and metabolic disturbances (higher values of basal insulin, glucose, triglycerides and cholesterol).

In the current population, subjective chronotype markers, such as central sleep timing and midpoint of intake, were delayed in more evening chronotypes as compared to more morning-types. Melatonin values at 01:00 h were significantly lower in evening-types than in morning-types, suggesting that melatonin may be still rising at those hours due to the later bedtime that characterizes evening-types.

In spite of being school-age children, with a marked schedule, we found differences in behaviors occurred mainly at night, when subjects were free to choose and endogenous trends appear. In general, more evening-types had increased physical activity in the evening while they had higher body temperature during the day which suggests an increase in sleepiness. This finding is consistent with previous studies that reported delayed physical activity patterns^25^ in evening-type children and presence of more episodes of daytime sleepiness^26^ in children with delayed behaviors. Considering that physical activity is an external synchronizer of the peripheral clocks^27^, these delayed behaviors may be per se affecting the circadian system function and may induce chronodisruption in these school-age children. Daytime sleepiness in youth has been associated with impairments in behavioral, mood, and performance domains^28^.

The duration of sleep plays an important role in school age children, given that sleep is relevant in maintaining good mental health and that short sleep has been associated with obesity^29^. As expected, sleep duration was decreased in evening-types. Previous studies performed in adolescents^8^ and preschool children^10^ have confirmed that late chronotypes have a decrease in sleep duration. Insufficient sleep has been associated with negative outcomes in several areas of health and functioning, including obesity, depression, school performance and risk-taking behavior^30^.

The “robustness” of daily sleep rhythm is determined by several parameters such as the relative amplitude, Circadian Function Index (CFI)^23^, interday stability and intraday variability. CFI provides information about the circadian system and facilitates objective evaluation of chronodisruption^15^. Lower CFI indicates less regular day-to-day rhythms, as demonstrated by a decrease in interday stability and in amplitude. In the current study, CFI of sleep was significantly lower among evening-types than morning-types, suggesting that evening-types have worse circadian function of sleep. Furthermore, evening-types had less depth of sleep, lower day–night contrast and more irregular habits.

In accordance with previous studies in adults^31^ and pre-school children^32^ findings of the present study show that evening-types experienced social jet lag more frequently than morning-types (7% and 3%, respectively). Social jet lag is a term describing misalignment between social and biological time^33^. Among evening chronotypes, schedules at school may interfere with individual sleep preferences and derive in chronodisruption^34^. During weekends, evening chronotypes are free to follow their biology and go to bed later and get up later in the morning. Social jet lag not only disrupts the amount of sleep, it also affects sleep quality, and irregular sleep is associated with poorer academic performance^35^. Social jet lag also affects circadian clocks and consequently the timing of hormones secretion, the activity of immune cells, and body temperature, and changes in mood at different times of day and night^36^ and has been shown to be a risk factor for psychological disorders^37^ and obesity^38^.

Light is the most important external synchronizer of the internal clock^39^. The timing of light exposure has a differential effect upon circadian phase. Early light exposure advances the cycle whereas late light delays circadian phase^40^. Results of the 7-day light pattern in the present study determined that evening-types presented a delayed light acrophase and lower values of light during the day. Later light acrophase was associated with approximately 1 h delay in the chronotype. Furthermore, light exposure during the last 2 h before bedtime (i.e., the timing in which melatonin starts to rise), it was 31% higher among evening-types than morning-types attaining values of 50 lx. Although there is considerable variation in individual response to light, it has been shown that light intensities of 30 lx are sufficient to suppress 50% of melatonin secretion^41^ and may produce a phase advance of more than three hours in the circadian pacemaker^40,42^. In the present study, when compared with morning types, evening types were exposed to light for shorter durations in the morning between wake time and school, which may also contribute to a more evening chronotype^43^. Similar findings have been reported among children in early years^44^.

Many studies relate eveningness to health problems^4, 45,46^. In adults, later chronotype is associated with greater morbidity, including higher rates of metabolic dysfunction and cardiovascular disease^4^ resulting in increased prevalence of metabolic syndrome, insulin resistance and sleep disturbances^5^. In the current study, central sleep timing was delayed approximately 1 h in evening-types. Previously, it has been reported that in children, each 1 h delay in chronotype is associated with more headaches, stomach and back aches, dizziness and worse self-rated health^45^. A 1 h delay in chronotype is also related to higher screen time and poor dietary habits^5^. Findings of the present study report that evening chronotypes have significantly higher values of basal insulin, glucose, triglycerides and cholesterol. In addition, and in agreement with previous studies performed in adolescents^7^ in the current population of school children, evening-types had a significantly higher BMI, which may be explained by several obesogenic behaviors, including insufficient sleep, less physical activity during the day and late eating^47^.

In previous studies, morningness has been positively related to intelligence, conscientiousness and learning objectives^48^. Early midpoint of sleep was associated with better grades^48^. Whereas late chronotypes are more idealistic, imaginative and intuitive^49^. These findings are in accordance with our results that evening-types had better grades in art, while no significant differences were found in other academic scores.

The authors present the following as limitations: (1) In the current study we detected metabolites in saliva and serum. The detection of metabolites in serum requires invasive techniques to extract a sample. Future studies might consider only detection of metabolites in saliva to avoid anxiety and stress to children because of blood extraction. (2) As an observational study conclusions of causality are limited.

Findings of the present study are a significant step in understanding chronotype and its relationship with chronodisruption and metabolic risk in children. The results show that in objectively assessed school age subjects, evening-types presented sleep alterations, social jet lag, more obesity, higher metabolic risk and better grades in art. Objective and non-invasive assessment of the individual chronotype, daily rhythms of sleep and circadian health should be included as part of a comprehensive approach to the pediatric patient. For children at risk, it is advisable to implement interventions to reduce eveningness, improve sleep and decrease social jet lag in order to decrease metabolic risk^50^.

## Data Availability

The datasets generated and/or analyzed during the current study are available from the corresponding authors on reasonable request.

## Abbreviations

TAP: Temperature (T), activity (A) and position (P)
IS: Interday stability
RA: Relative amplitude
CFI: Circadian function index
ONTIME-Jr: Obesity, nutrigenetics, timing and mediterranean, junior
MCTQ: Munich chronotype questionnaire
BMI: Body mass index
