# Author Correction: Evening types have social jet lag and metabolic alterations in school-age children

**DOI:** 10.1038/s41598-021-84775-9

**Published:** 2021-03-03

**Authors:** Nuria Martínez-Lozano, Gloria Maria Barraco, Rafael Rios, Maria José Ruiz, Asta Tvarijonaviciute, Paul Fardy, Juan Antonio Madrid, Marta Garaulet

**Affiliations:** 1 Department of Physiology, University of Murcia, IMIB-Arrixaca, 30100 Murcia, CP Spain; 2 Health Area of Lorca, Lorca, Murcia Spain; 3 Division of Sleep and Circadian Disorders, Departments of Medicine and Neurology, Brigham and Women’s Hospital, Boston, MA 02115 USA

Correction to: *Scientific Reports* 10.1038/s41598-020-73297-5, published online 07 October 2020

The Acknowledgements section in this Article is incomplete.

“We thank all children and their families and the three schools Maristas, San Antonio and San Pablo CeI for their help and support, specially to Mari Carmen Blaya, Jose Ignacio Peña and María José Martínez.”

should read:

“We thank all children and their families and the three schools Maristas, San Antonio and San Pablo CeI for their help and support, specially to Mari Carmen Blaya, Jose Ignacio Peña and María José Martínez. This work has been supported in part by The Spanish Government of Investigation, Development and Innovation (SAF2017-84135-R) including FEDER co-funding. The Autonomous Community of the Region of Murcia through the Seneca Foundation (20795/PI/18) and NIDDK R01DK105072 granted to M. Garaulet”

